# Limits of rapid diagnostics: genomic and structural insights into OXA-48–like mediated carbapenem resistance in *Escherichia coli*

**DOI:** 10.3389/fmicb.2026.1790597

**Published:** 2026-04-02

**Authors:** Aya A. Diab, Deena Jalal, Youssef M. Fadel, Omar Samir, Lobna Shalaby, Mervat Elanany, Sabah A. Abo-Elmaaty, Ramy K. Aziz, Ahmed AbdelAziz Sayed, Mervat G. Hassan

**Affiliations:** 1 Basic Research Department, Children’s Cancer Hospital Egypt 57357, Cairo, Egypt; 2 Bioinformatics Group, Center for Informatics Science, School of Information Technology and Computer Science, Nile University, Giza, Egypt; 3 Infectious Disease Unit, Children’s Cancer Hospital Egypt 57357, Cairo, Egypt; 4 Department of Pediatric Oncology, National Cancer Institute, Cairo University, Cairo, Egypt; 5 Microbiology Unit, Children’s Cancer Hospital Egypt 57357, Cairo, Egypt; 6 Department of Clinical Pathology, Faculty of Medicine, Cairo University, Cairo, Egypt; 7 Botany and Microbiology Department, Faculty of Science, Benha University, Al Qalyubiyah, Egypt; 8 Department of Microbiology and Immunology, Faculty of Pharmacy, Cairo University, Cairo, Egypt; 9 Department of Biochemistry, Faculty of Science, Ain Shams University, Cairo, Egypt

**Keywords:** bioinformatics, carbapenem resistance, genomics, OXA-48 variants, protein structural modeling

## Abstract

Carbapenem-resistant *Escherichia coli* (CREC) represent a major clinical threat because of limited treatment options and frequent multidrug resistance. While rapid molecular diagnostics improve treatment outcomes, the detection of carbapenemase genes, such as *bla*_OXA-48_-like, does not always correspond to phenotypic resistance, complicating management of bloodstream infections. We investigated 20 bloodstream *E. coli* isolates, from pediatric cancer patients at Children’s Cancer Hospital Egypt 57357, that harbored *bla*_OXA-48_-like genes yet were phenotypically susceptible to meropenem. Whole-genome sequencing was performed to characterize resistomes, virulomes, plasmid content, and the genetic context of OXA-48–like variants. The isolates belonged to 12 sequence types (STs), with ST405 and ST410 being the most frequent. *bla*_OXA-244_ predominated and was primarily chromosomal, whereas *bla*_OXA-181_ and *bla*_OXA-484_ were plasmid-borne, often co-localized with *qnrS1*. Resistome and virulome profiles were broadly conserved across meropenem-susceptible and resistant isolates. Structural modeling and protein–ligand interaction analyses of OXA-48, OXA-244, OXA-181, and OXA-484 illustrate how the substitutions Arg214Gly and Thr104Ala are consistent with localized alterations in active site geometry, despite preservation of key ligand interactions. These observations help explain the observed discordance between genotype and phenotype and highlight the limitations of relying solely on rapid gene detection for treatment decisions. Collectively, these findings highlight the need to interpret rapid PCR-based carbapenemase detection in conjunction with phenotypic susceptibility testing and genomic context, supporting balanced antimicrobial decision-making that preserves carbapenem use when appropriate while maintaining vigilance against the silent spread of carbapenemase-producing strains.

## Graphical abstract

**Figure:**
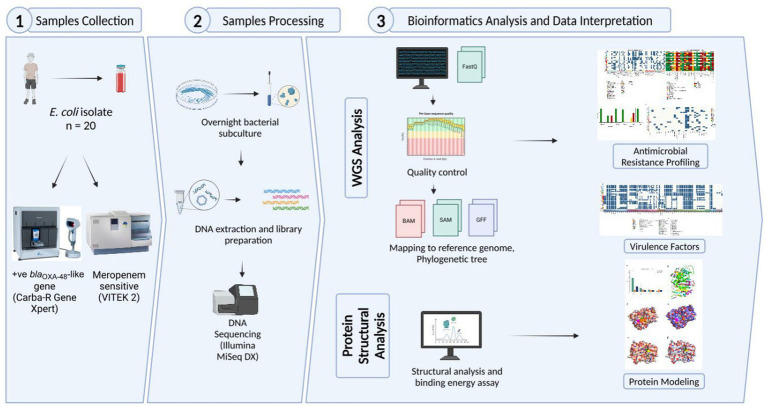
A three-step workflow: (1) Sample collection involves isolating *E. coli* (*n* = 20), and testing for OXA-48–like genes using Carba-R Gene Xpert and antimicrobial resistance using VITEK® 2. (2) Sample processing includes bacterial subculture, DNA extraction, library preparation, and sequencing using Illumina® MiSeq DX. (3) Bioinformatics analysis is subdivided into (i) WGS analysis, which encompasses quality control, genome mapping, antimicrobial resistance profiling, and virulence factor; and (ii) protein structural analysis, which encompasses structure prediction and binding energy assays.

## Introduction

Carbapenems are a class of beta-lactam antibiotics characterized by a beta-lactam ring fused to an unsaturated five-membered ring in which a carbon atom replaces sulfur. This unique structure provides the carbapenems with enhanced stability and resistance to most beta-lactamases, including extended spectrum beta-lactamases (ESBLs; Elshamy and Aboshanab, 2020). Carbapenems gained their prominence from their broad-spectrum activity against both Gram-positive and Gram-negative bacteria, including some multidrug-resistant bacteria, such as *Escherichia coli* (Papp-Wallace et al., 2011; El-Gamal et al., 2017; Bassetti et al., 2009). Currently, the carbapenems available in the pharmaceutical market are meropenem, imipenem, ertapenem, and doripenem. Because of its combined broad-spectrum activity, favorable safety profile, and short half-life that allows flexible dosing schedules, meropenem is considered the preferred carbapenem treatment option (Elshamy and Aboshanab, 2020; Shien Lo et al., 2012; Tamma et al., 2022). The most common mechanisms of carbapenem resistance in Enterobacterales is the expression of carbapenemase enzymes, whose encoding genes can be transferred among members of the Enterobacterales through mobile genetic elements (Tilahun et al., 2021). The most frequently identified genes encoding carbapenemases are class A KPC, class B metallo-*β*-lactamases (IMP, VIM and NDM) and class D OXA-type enzymes (OXA-48-like) (Dagher et al., 2018). In addition, carbapenem resistance can arise from the combined presence of AmpC enzymes or ESBLs with reduced outer membrane permeability, most commonly mediated by mutations or loss of outer membrane porins (Hamzaoui et al., 2018; Larkin et al., 2020; Wong et al., 2019).

Traditionally, the treatment of carbapenem-resistant Enterobacterales bloodstream infections (CRE-BSIs) relied primarily on time-consuming phenotypic antimicrobial susceptibility testing and carbapenem minimum inhibitory concentrations (MICs). High-dose or prolonged-infusion carbapenems for isolates with lower MICs, often combined with a second active agent such as an aminoglycoside or polymyxin (Madney et al., 2025). The introduction of rapid molecular diagnostics, such as PCR-based detection, represented a major advance in CRE-BSI management by enabling early detection of carbapenemase genes directly from blood cultures (Ziad et al., 2025). Identification of specific carbapenemases, such as KPC, NDM, or OXA-48-like enzymes, increasingly informed early therapeutic escalation to targeted *β*-lactam/*β*-lactamase inhibitor combinations, including ceftazidime–avibactam, often before complete phenotypic susceptibility data became available. This approach has been associated with improved timeliness of appropriate therapy and better clinical outcomes in multiple studies (Banerjee and Humphries, 2017; Satlin et al., 2022; Jankowski, 2021). However, the clinical utility of rapid gene-based diagnostics depends on the assumption that detection of a carbapenemase gene reliably predicts functional resistance.

Although ceftazidime–avibactam is a generally effective and well tolerated treatment, unnecessary use can exert selective pressure that drives rapid resistance, including *bla*_KPC_ mutations and acquisition of metallo-*β*-lactamases such as NDM, which are not inhibited by avibactam (Perez et al., 2016; Liu et al., 2025). Exposure in high-burden hospital environments or immunocompromised patients has been linked to plasmid-mediated resistance evolution (Jalal et al., 2025). Consequently, premature escalation to ceftazidime–avibactam when carbapenems remain active may inadvertently promote the emergence and dissemination of more difficult-to-overcome resistance mechanisms.

Carbapenem-resistant *E. coli* (CREC) have emerged as a major global health threat owing to resistance to last-line carbapenems and frequent multidrug co-resistance (Luo et al., 2018; Huang et al., 2024). In 2017, the World Health Organization classified CREC among the highest-priority pathogens requiring urgent development of new therapeutic options (Huang et al., 2024). In addition to antimicrobial resistance, CREC often possess virulence-associated factors that facilitate bloodstream invasion, contributing to severe infections such as bacteremia and septicemia, and resulting in increased morbidity, mortality, and healthcare burden (Dagher et al., 2018; Huang et al., 2024; Slown et al., 2022; Jomehzadeh et al., 2022).

At the Children’s Cancer Hospital Egypt 57357 (CCHE 57357), the implementation of rapid diagnostics has significantly accelerated treatment initiation and improved patient care (Madney et al., 2025). However, a clinical challenge has emerged regarding the correlation between molecular and phenotypic resistance profiles in some isolates. Our surveillance of carbapenem-resistant Enterobacterales (CRE) isolates revealed that approximately 27% (*n* = 53/189) harbored *bla*_OXA-48_ as the sole carbapenemase gene (Jalal et al., 2025). Nearly half of these isolates demonstrated preserved phenotypic susceptibility to meropenem despite being resistant to ertapenem. Under current rapid-diagnostic protocols, patients infected with these bacteria are often escalated to ceftazidime–avibactam–based therapy based on gene detection alone, despite the retained phenotypic activity of meropenem.

In this study, we applied whole-genome sequencing to systematically and comprehensively investigate the molecular basis of discordance between rapid molecular detection of carbapenemase genes and preserved phenotypic susceptibility to meropenem in a subset of carbapenemase-producing *E. coli* bloodstream isolates. Specifically, we sought to determine whether genetic variation within OXA-48-like enzymes and associated genomic features could explain the observed susceptibility patterns and contribute to bloodstream infection potential. By integrating phenotypic data with genomic and protein structural analyses, we aimed to clarify the limitations of gene-based diagnostics in guiding antimicrobial therapy and inform more precise treatment strategies for CRE bloodstream infections.

## Materials and methods

### Ethical approval

The Institutional Review Board at CCHE 57357 approved the study following ICH GCP guidelines and local and institutional regulations.

### Sample collection and antimicrobial susceptibility testing

From the initial cohort of 189 CRE isolates, we focused on a specific subset of 20 *E. coli* isolates that tested positive for *bla*_OXA-48_ via the Cepheid Xpert Carba-R assay but demonstrated phenotypic susceptibility (or intermediate susceptibility) to meropenem by disc diffusion to clarify the molecular mechanisms underlying this discrepancy. Antimicrobial susceptibility testing (AST) was carried out using the Vitek 2 AST cards GN222 (bioMérieux SA, Marcy l’Etoile, France) following the manufacturer’s protocols. Results interpretation was conducted in accordance with the guidelines established by the Clinical and Laboratory Standards Institute (CLSI; Clinical and Laboratory Standards Institute (CLSI), 2023). Based on CLSI breakpoints, isolates with MICs of ≤1 μg/mL were considered sensitive, those with MICs of 2 μg/mL were intermediate, and isolates with MICs ≥4 μg/mL were classified as resistant, providing clear criteria for susceptibility interpretation.

### DNA extraction and whole genome sequencing

For each sample, a single colony was inoculated in 2 mL Luria-Bertani (LB) medium then incubated overnight at 37 °C in a shaking incubator. The samples were centrifuged at 14,000 × g for 10 min to pellet the bacterial cells. DNA was then extracted using the PureLink™ Microbiome DNA Purification Kit following the manufacturer’s instructions (Thermo Fisher Scientific, United States). The library preparation was performed using Nextera® XT DNA Library Preparation Kit and Nextera® XT Index Kit by Illumina according to the manufacturer’s instructions (Illumina, United States). The libraries were then normalized, pooled and subjected to 300-base paired-end read sequencing using an Illumina MiSeqDx system according to the manufacturer’s protocol (Jalal et al., 2021).

### Bioinformatics analysis

**Figure 1:**
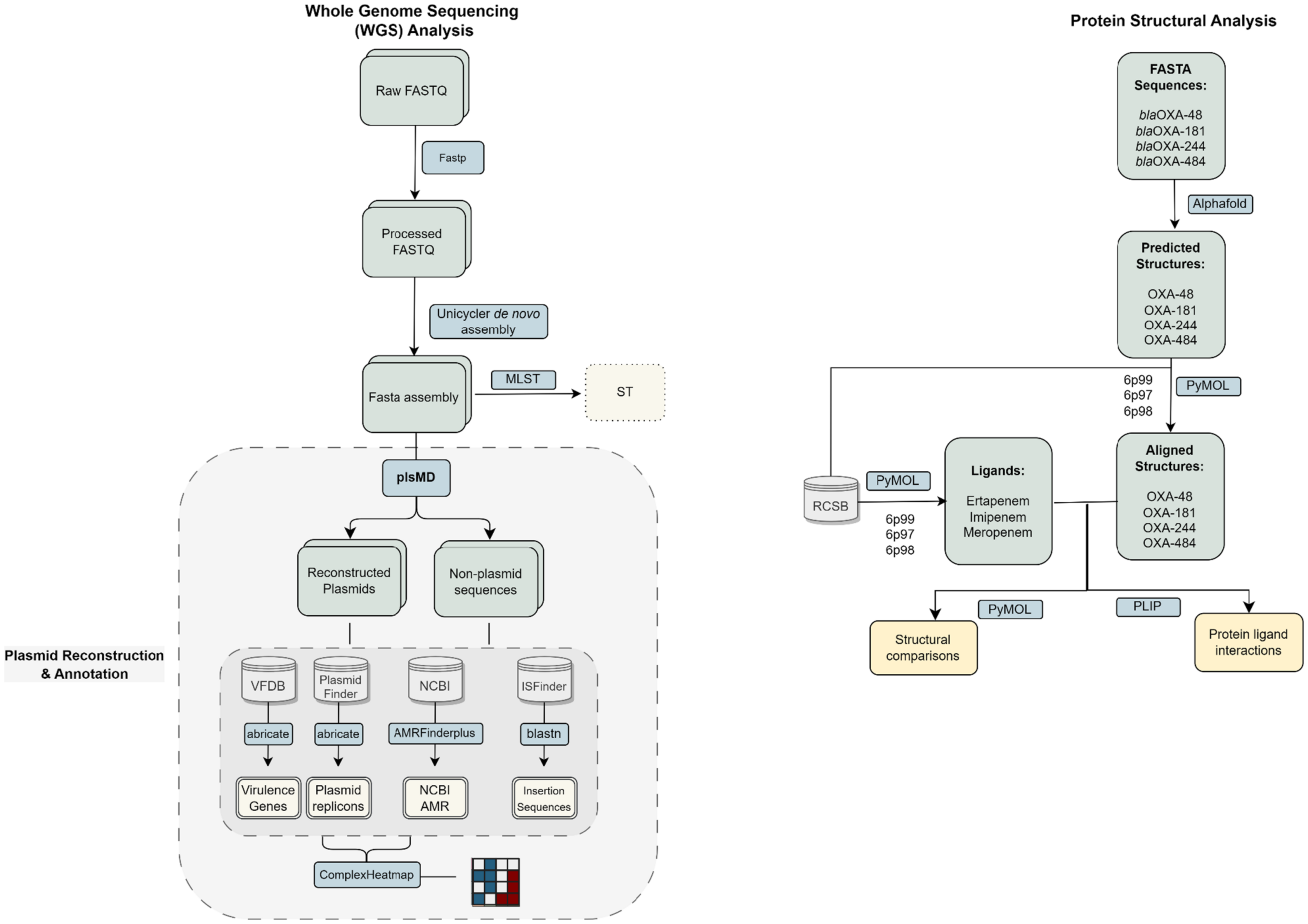
Flowchart illustrates the bioinformatics pipeline used to analyse whole genome sequencing data, including preprocessing (Fastp), assembly (Unicycler, bwa-mem), and annotation (Abricate, AMRFinderPlus) to identify MLST, plasmids, virulence, and AMR genes.

The bioinformatics pipeline (Figure 1) was previously described (Jalal et al., 2025) and is summarized in Supplementary Figure 1. Below is a brief summary of the pipeline.

#### a. Whole-genome sequencing (WGS) assembly and multiple locus sequence typing (MLST)

Read pairs were quality filtered, and adapter sequences were removed by fastp (Chen et al., 2018). Unicycler (Wick et al., 2017) was used to *de novo* assemble the merged read. Sequence types were determined by database (https://pubmlst.org/; accessed on January 1, 2023; Jolley and Maiden, 2010) following the Achtmann scheme for *E. coli*.

#### b. Identification of resistome, virulome, and mobile genetic elements

plsMD (Lotfi et al., 2025) was used for reconstructing full-length plasmid sequences and extracting them from the remaining chromosomal contigs. Both were analyzed to identify AMR genes against the AMRfinderPlus database (Feldgarden et al., 2021), virulence factor (VF) genes with VFDB (Chen et al., 2005), insertion sequences (ISs) with ISfinder (Siguier et al., 2006), plasmid replicons using Plasmidfinder tool (Carattoli et al., 2014), and relaxase types using MOB-typer (Robertson and Nash, 2018).

#### c. Protein structural analysis

The PDB structures 6P97, 6P98, and 6P99 were retrieved from the RCSB Protein Data Bank (Smith et al., 2019), and their co-crystallized ligands (imipenem, meropenem, and ertapenem, respectively) were isolated using PyMOL^1^ and saved as separate files. The 6p98, 6p97, and 6p99 PDB structures were retrieved from the RCSB PDB database. AlphaFold (Jumper et al., 2021) was used to computationally generate the protein structures of OXA-48, OXA-181, OXA-244, and OXA-484. To study ligand interactions, the AlphaFold-generated OXA-48 structure was aligned to the experimental structures (6P97–6P99) in PyMOL, and the corresponding ligands were subsequently transferred to the aligned model and saved as protein–ligand complexes. Protein–ligand interactions were analyzed by the PLIP web service (Adasme et al., 2021). Structural comparisons between the different protein variants were performed with the PyMOL Molecular Graphics System, Version 2.5.2 (Schrödinger, LLC) for visualization and analysis.

## Results

### OXA-48-like variants across several STs in *Escherichia coli*

Using the Oxford scheme, we categorized all 20 isolates into 12 distinct STs. The predominant ST was ST405 (four samples), followed by ST410 (three samples), and then ST10, ST131, and ST361 (two samples each). Each of the remaining isolates belonged to a different type (ST1139, ST155, ST167, ST3268, ST4981, ST501 and ST69).

### Phenotypic susceptibility despite carbapenemase presence

Upon examining the presence of *bla*_OXA-48_ in the isolates, we observed that all 20 genomes harbored *bla*_OXA-48_-like genes, including *bla*_OXA-244_, *bla*_OXA-181_, and *bla*_OXA-484_, which were associated with varying resistance profiles. *bla*_OXA-244_ was the most frequently detected variant, present in the majority of samples (*n* = 15), while *bla*_OXA-181_ was identified in four isolates (A01, A06, A15, and A17). A single isolate (A08) carried *bla*_OXA-484_ (Table 1 and Supplementary Figure 1).

**Table 1:** Distribution and genomic location of OXA carbapenemase variants among study isolates.

Isolate	MLST	bla_OXA-48_-like variant	Genomic location	Genetic context	Assembly size and depth	Reconstructed plasmid size and number of contigs	Meropenem MIC (mg/L)—interpretation	Mobility features	Replicon type
A01	405	*bla*_OXA-181_	Plasmid		12,503 bp; 3.79x	69,261 bp (43 contigs)	1–S	MOBF relaxase, MPF_F, conjugative	rep_cluster_1195/IncFIC(FII)/ColKP3
A02	501	*bla*_OXA-244_	Chromosome	IS10A–IS4	1,774 bp; 2.47x	–	≤0.5–S		
A03	1,139	*bla*_OXA-244_	Chromosome	IS10A–IS4	1,769 bp; 3.05x	–	1–S		
A04	410	*bla*_OXA-244_	Chromosome	IS10A–IS4	1,774 bp; 2.15x	–	≤0.5–S		
A05	69	*bla*_OXA-244_	Chromosome	–	2,233 bp; 1.48x	–	1–S		
A06	405	*bla*_OXA-181_	Plasmid		8,568 bp; 3.69x	70,196 bp (42 contigs)	≤0.5–S	MOBF relaxase, MPF_F, conjugative	rep_cluster_1195/IncFIC(FII)/ColKP3
A07	10	*bla*_OXA-244_	Chromosome	IS10A–IS4	1,774 bp; 3.41x	–	≤0.5–S		
A08	10	*bla*_OXA-484_	Plasmid		12,563 bp; 1.88x	51,366 bp (5 contigs)	≤0.5–S	MOBP relaxase, MPF_T, conjugative	rep_cluster_1195/IncX3/ColKP3
A09	405	*bla*_OXA-244_	Chromosome	–	2,231 bp; 2.48x	–	1–S		
A10	361	*bla*_OXA-244_	Chromosome	IS10A–IS4	1,767 bp; 1.23x	–	1–S		
A11	–	*bla*_OXA-244_	Chromosome	IS10A–IS4	1,774 bp; 1.82x	–	2–I		
A12	361	*bla*_OXA-244_	Chromosome	IS10A–IS4	1,767 bp; 3.95x	–	–S		
A13	131	*bla*_OXA-244_	Chromosome	IS10A–IS4	1,773 bp; 1.67x	–	≤0.25–S		
A14	131	*bla*_OXA-244_	Chromosome	IS10A–IS4	1,774 bp; 0.49x	–	1–S		
A15	405	*bla*_OXA-181_	Plasmid		8,568 bp; 3.65x	70,969 bp (16 contigs)	1–S	MOBF relaxase, MPF_F, conjugative	rep_cluster_1195/IncFIC(FII)/ColKP3
A16	4,981	*bla*_OXA-244_	Chromosome	IS10A–IS4	1,772 bp; 1.40x	–	≤0.5–S		
A17	155	*bla*_OXA-181_	Plasmid		8,567 bp; 3.23x	71,144 bp (62 contigs)	≤0.5–S	MOBF relaxase, MPF_F, conjugative	rep_cluster_1195/IncFIC(FII)/ColKP3
A18	167	*bla*_OXA-244_	Chromosome	IS10A–IS4	1,767 bp; 3.74x	–	1–S		
A19	3,268	*bla*_OXA-244_	Chromosome	–	2,231 bp; 1.30x	–	≤0.5–S		
A20	131	*bla*_OXA-244_	Chromosome	IS10A–IS4	1,785 bp; 2.91x	–	≤0.5–S		

To contextualize these findings, we compared the carbapenemase gene content of the meropenem-susceptible isolates with that of meropenem-resistant isolates (*n* = 169) collected during the same surveillance period from the same hospital as part of a previously characterized CRE bloodstream infection cohort (Jalal et al., 2025). Notably, none of the meropenem-susceptible isolates carried other carbapenemase genes conferring high-level resistance to carbapenems, such as *bla*_NDM_, which were restricted to meropenem-resistant strains in the larger cohort (Supplementary Figure 1 and Jalal et al., 2025).

### Similar resistomes and virulomes across meropenem-susceptible and resistant isolates

The majority of resistance determinants identified in the previously characterized CRE cohort, including *β*-lactamase genes (*bla*_EC_, *bla*_TEM-1_, *bla*_CTX-M-15_), quinolone resistance mechanisms mediated by *gyrA/parC* mutations and *qnrS1*, aminoglycoside-modifying enzymes, sulfonamide resistance genes (*sul1/sul2*), macrolide resistance genes, and multidrug efflux systems, were also detected in the meropenem-susceptible isolates in this study, indicating a shared resistome background (Supplementary Figure 1 and Jalal et al., 2025).

Similarly, virulence profiling revealed no major qualitative differences between the meropenem-susceptible and meropenem-resistant isolates. Core virulence gene clusters involved in adhesion, biofilm formation, iron acquisition, and interbacterial competition, including *fim*, *csg*, *ent*, *yag/ecp*, *ybt*, and type VI secretion system components, were conserved across both phenotypes, consistent with comparable invasive potential in bloodstream infections (Supplementary Figure 2 and Jalal et al., 2025).

Collectively, these data indicate that phenotypic meropenem resistance in this population is primarily driven by the acquisition of highly active carbapenemases rather than broad differences in AMR gene content or virulence gene repertoires, supporting the presence of shared resistomes and virulomes between meropenem-susceptible and -resistant lineages.

### OXA-48–like genes are predominantly chromosomal across *Escherichia coli* sequence types

**Figure 2:**
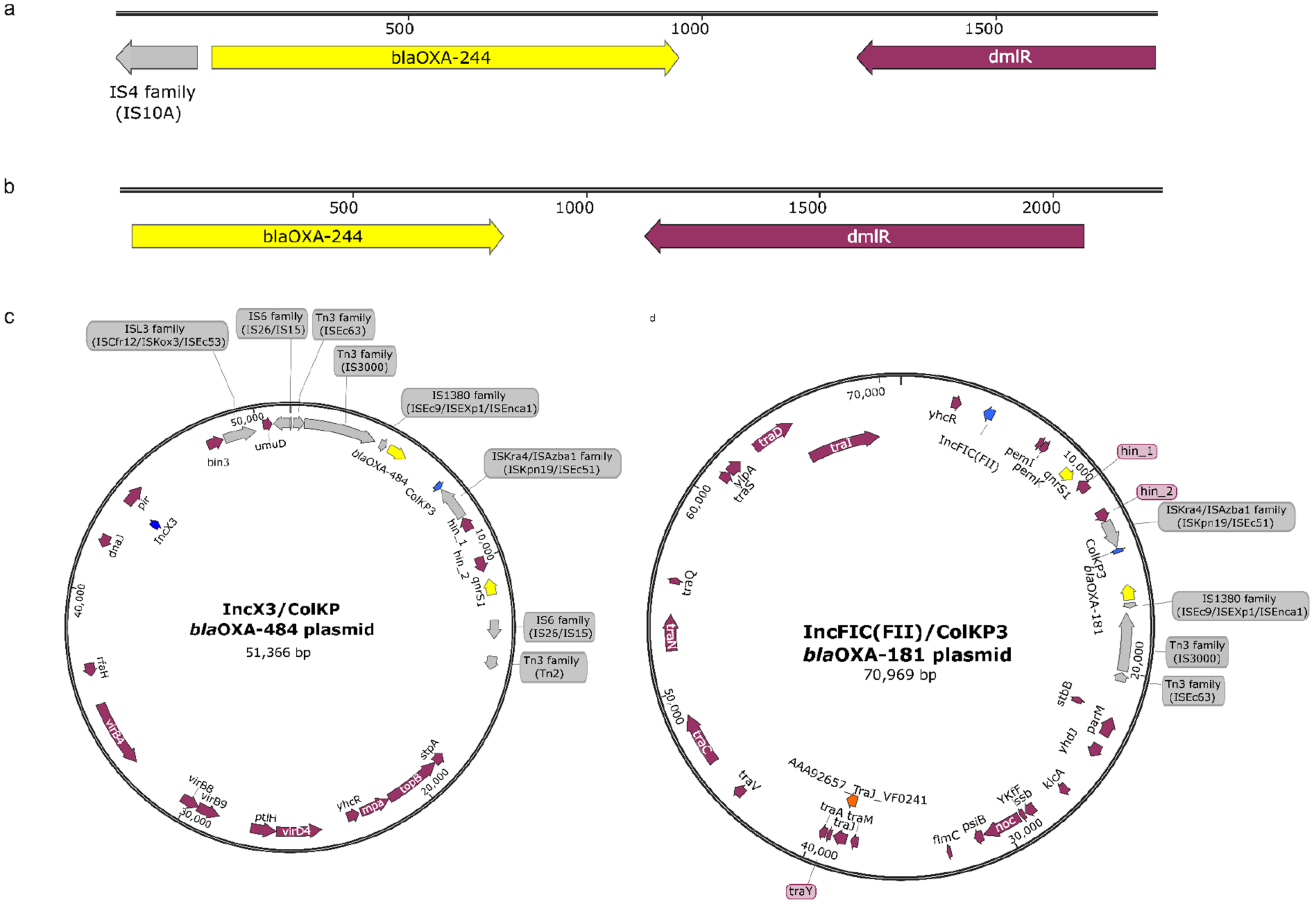
Genomic context of *bla*_OXA-48_-like variants in *E. coli*. SnapGene software (URL: https://www.snapgene.com) was used to visualize the genetic environments of *bla*_OXA-48_-like genes. **(a,b)** Chromosomal *bla*_OXA-244_ in multiple STs, typically flanked by a single upstream IS10A element. **(c)** Plasmid-borne *bla*_OXA-181_ on IncFIC(FII)/ColKP3 plasmids with *qnrS1*. **(d)** Plasmid-borne *bla*_OXA-484_ on IncX3/ColKP3 plasmids with *qnrS1*. Arrows indicate gene orientation: yellow, AMR genes; blue, replicon types; purple, other genes; grey, insertion sequences.

To determine the genomic context of the *bla*_OXA-48_-like genes, we used plsMD, which separates plasmid-derived sequences from chromosomal contigs and reconstructs complete plasmid sequences that are otherwise difficult to resolve from short-read assemblies. Despite the overall similarity in AMR gene content, the different *bla*_OXA-48_-like variants exhibited distinct genetic contexts. *bla*_OXA-244_ was found predominantly integrated into the chromosome across multiple *E. coli* STs, including major STs such as ST410, ST405, ST361, and ST131, with lower prevalence in ST167. Because short-read assemblies frequently break at repetitive regions, such as insertion sequences and transposable elements, we examined the contigs harboring *bla*OXA-244 to infer associated mobilizable elements. In most isolates, *bla*OXA-244 was associated with a single upstream IS10A element without a corresponding downstream insertion sequence, and a few isolates lacked any insertion sequences entirely. This configuration suggests a remnant transposition event followed by chromosomal stabilization, consistent with limited ongoing mobility of *bla*_OXA-244_. Genetic maps of the contigs harboring *bla*_OXA-244_ are presented in Figures 2a,b.

In contrast, *bla*_OXA-181_ and *bla*_OXA-484_ was plasmid-borne as identified by the reconstructed plasmids using plsMD (Figures 2c,d). *bla*_OXA-181_ was found residing on IncFIC(FII)/ColKP3 replicon-type plasmids and co-harbored with the quinolone resistance gene *qnrS1* (Figure 2c). Similarly, *bla*_OXA-484_ was identified on IncX3/ColKP3 replicon-type plasmids, also in association with *qnrS1* (Figure 2d). This consistent co-localization suggests co-transfer of carbapenemase and quinolone resistance determinants, suggesting quinolone exposure as a potential selective driver for the dissemination and maintenance of low-level carbapenem resistance in *E. coli*. Details of the genetic environment surrounding blaOXA-48-like genes, including gene location, assembly contig length and depth, reconstructed plasmid length and number of contigs, and replicon/relaxase types, are summarized in Table 1.

### Active site conformational differences among OXA-48-like variants

To examine structural features that may contribute to the observed phenotypic susceptibility profiles, we performed comparative structural modeling of OXA-48 and its variants (OXA-181, OXA-244, and OXA-484) in complex with meropenem, imipenem, and ertapenem. As crystallographic structures are available only for OXA-48, AlphaFold was used to model all variants, including OXA-48 itself, to ensure methodological consistency and minimize modeling-related bias. To validate the modeled structures, we analyzed ligand interactions of OXA-48 with meropenem, imipenem, and ertapenem (Supplementary Figure 1) using ligands extracted from published crystal structures (PDB IDs: 6P98, 6P97, and 6P99, respectively). The predicted interactions were highly concordant with crystallographic data, involving conserved residues such as Ser70, Ser118, Val120, Leu158, Lys208, Tyr211, and Arg250. Additional hydrophobic contacts involving Leu247 and Gly248 were observed for ertapenem, consistent with its distinct side-chain chemistry.

**Figure 3:**
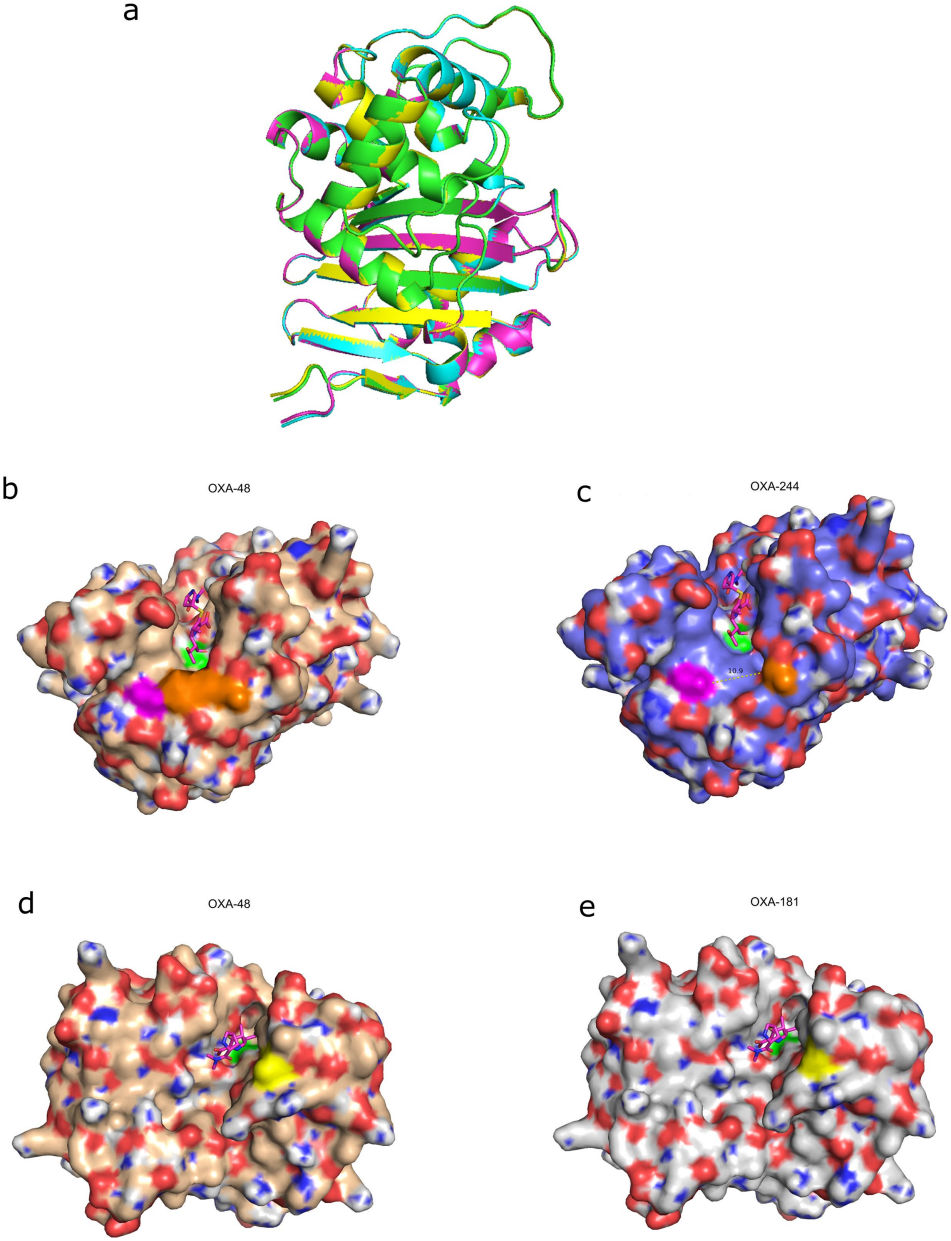
Changes in active site architecture underlie the reduced carbapenemase activity in OXA-48 variants. **(a)** Overlay of OXA-48, OXA-181, OXA-244, and OXA-484 backbone structures, demonstrating identical overall conformations. **(b,c)** Surface structures of OXA-48 and OXA-244 with meropenem bound in the active site. Active site Ser70 is highlighted in green, Gln124 in magenta, and residue 214 is shown in orange (arginine in OXA-48 and glycine in OXA-244). **(d,e)** Surface structures of OXA-48 and OXA-181 with meropenem bound in the active site. Residue 104 is highlighted in yellow (threonine in OXA-48 and alanine in OXA-181).

Overlay of the backbone structures of OXA-48 and its variants revealed no global conformational differences (Figure 3a). However, localized structural changes were evident in the active site region. In OXA-244 and OXA-484, the Arg214Gly substitution resulted in widening of the active site groove between residues 214 and 124, increasing the distance from approximately 4.5 Å in OXA-48 to 10.5 Å (Figures 3b,c). In addition, OXA-181 and OXA-484 harbor a Thr104Ala substitution, which introduced a subtle structural rearrangement adjacent to the active site (Figures 3d,e). While these substitutions did not markedly alter direct ligand–residue contacts in our models, they modified the geometry of the active site and its surrounding region. These structural differences are consistent with previously reported biochemical studies demonstrating reduced catalytic efficiency of OXA-244 and OXA-484 relative to OXA-48 and OXA-181, particularly against meropenem and ertapenem, as well as substrate-dependent effects on imipenem hydrolysis (Rima et al., 2021; Gonzalez et al., 2024). Together, these observations indicate that localized active site architecture, rather than gross structural rearrangements, is associated with the phenotypic susceptibility patterns observed in isolates carrying OXA-48–like variants (Smith et al., 2019; Docquier et al., 2009).

## Discussion

The global spread of CREC represents a critical public health threat because of their high burden of resistance, limited treatment options, and potential for widespread transmission (Huang et al., 2024). Their dissemination, facilitated by interactions across human, animal, and environmental reservoirs, poses severe challenges for clinical control. Early detection of carbapenem resistance is essential for appropriate treatment, particularly in vulnerable patients. Rapid PCR-based diagnostics have become an integral part of routine care, facilitating faster treatment initiation and improved outcomes; however, contradictory results between phenotypic and PCR-based methods complicate interpretation and management.

In this study, we provide mechanistic insight into one major contributor to genotype–phenotype discordance by identifying specific OXA-48-like variants, OXA-244, OXA-181, and OXA-484, which exhibit attenuated meropenem-hydrolyzing activity. Notably, *bla*_OXA-244_ was the most prevalent variant in our cohort and was found integrated into the chromosome across multiple STs. This chromosomal localization within transposable elements has been reported in several organisms (Pedraza et al., 2022) and suggests a shift toward stable inheritance. This variant arises from a single-point mutation in the *bla*_OXA-48_ gene, resulting in an Arg214Gly substitution in OXA-244 (Rima et al., 2021; Hoyos-mallecot et al., 2017; Masseron et al., 2020). While *bla*_OXA-181_ is traditionally associated with IncX3 plasmids through specific insertion sequences (Qin et al., 2018; Pitout et al., 2020), we found it associated with IncFIC(FII)/ColKP3 replicon type plasmids in our isolates, highlighting regional genomic diversity. In contrast, the *bla*_OXA-484_ variant, which is closely related to *bla*_OXA-181_ but carries the Arg214Gly substitution in OXA-484, remained exclusively associated with IncX3 plasmids (Gonzalez et al., 2024; Findlay et al., 2017; Ramsamy et al., 2022; Hooban et al., 2022; Findlay et al., 2023; Ge et al., 2023; Yu et al., 2022). The inferred genomic locations and surrounding mobile elements are based on plasmid reconstructions generated by plsMD, and therefore depend on the accuracy of short-read–based plasmid assembly. Although plsMD demonstrates strong performance in reconstructing plasmid sequences (Lotfi et al., 2025), long-read sequencing would be required to fully resolve plasmid structures and confirm the precise genetic contexts.

To align our observations with established structural data, we performed a structural comparison of OXA-48 and its variants to assess whether variant-specific substitutions were associated with localized changes in active site architecture. Although the overall backbone structures remain highly conserved, our modeling highlights variant-specific alterations in the active site region. Specifically, the Arg214Gly substitution in OXA-244 and OXA-484 substantially widens the active site groove, consistent with the established role of the β5–β6 loop and Arg214 in substrate recognition (Smith et al., 2019; Docquier et al., 2009; Oueslati et al., 2020). This principle is further supported by the loop-deficient OXA-163 variant, which has been shown to exhibit impaired catalytic activity (Stojanoski et al., 2021). In addition, the Thr104Ala substitution present in OXA-181 and OXA-484 introduces a more subtle conformational change adjacent to the active site, which may influence substrate accommodation (Docquier et al., 2009). Although this study is limited by reliance on in silico modeling, where functional effects can be influenced by gene expression, plasmid copy number, or other cellular factors, and by a relatively small number of isolates, our observations align with previously published biochemical studies (Gonzalez et al., 2024; Oueslati et al., 2020; Hirvonen et al., 2021). Specifically, those studies report reduced and substrate-dependent catalytic efficiencies for OXA-244 and OXA-484 relative to OXA-48 and OXA-181, (*k*_cat_/*K*_m_ for meropenem: OXA-48 6 mM^−1^·s^−1^, OXA-181 1.5 mM^−1^·s^−1^, OXA-484 0.2 mM^−1^·s^−1^, OXA-244 0.8 mM^−1^·s^−1^) supporting the biological relevance of the structural changes we observed (Gonzalez et al., 2024; Oueslati et al., 2020; Hirvonen et al., 2021).

While these results may seem to favor phenotypic assays over rapid PCR, the detection of attenuated variants remains a vital clinical issue. Relying solely on meropenem susceptibility could allow the silent spread of these variants, a risk that has also been reported in other carbapenem-resistant organisms (Pedraza et al., 2022). Although these isolates demonstrate *in vitro* susceptibility, they may exhibit clinical resistance due to the high inoculum often seen in bloodstream infections. Additionally, the presence of these genes represents a “pre-resistant” state, where minor secondary mutations, such as porin loss, can rapidly enhance resistance under selective pressure. Consequently, the clinical decision to escalate to ceftazidime-avibactam in these cases serves as an appropriate risk-mitigation strategy to ensure bactericidal activity in immunocompromised patients. In this context, rapid molecular diagnostics may aid early detection; alternatively, reduced susceptibility to ertapenem may provide an additional phenotypic indicator of OXA-48-like carbapenemases, as these enzymes often hydrolyze ertapenem more efficiently than other carbapenems. Altogether, this study addresses the commonly observed paradox of genotype–phenotype discordance in detecting carbapenemase-mediated resistance and emphasizes the need for diagnostic assays capable of distinguishing between high-activity and attenuated OXA-48-like variants to support precise treatment decisions.

## Data Availability

All data generated and analyzed during this study are included in this article and published online on NCBI with the SRA accession number PRJNA1209707.

## Footnotes

1 https://pymol.org.
