# Supplementary figures and images for: Limits of rapid diagnostics: genomic and structural insights into OXA-48–like mediated carbapenem resistance in Escherichia coli
Source: Front Microbiol. 2026 Apr 2;17:1790597. doi: 10.3389/fmicb.2026.1790597 (PMC13085321; doi:10.3389/fmicb.2026.1790597)

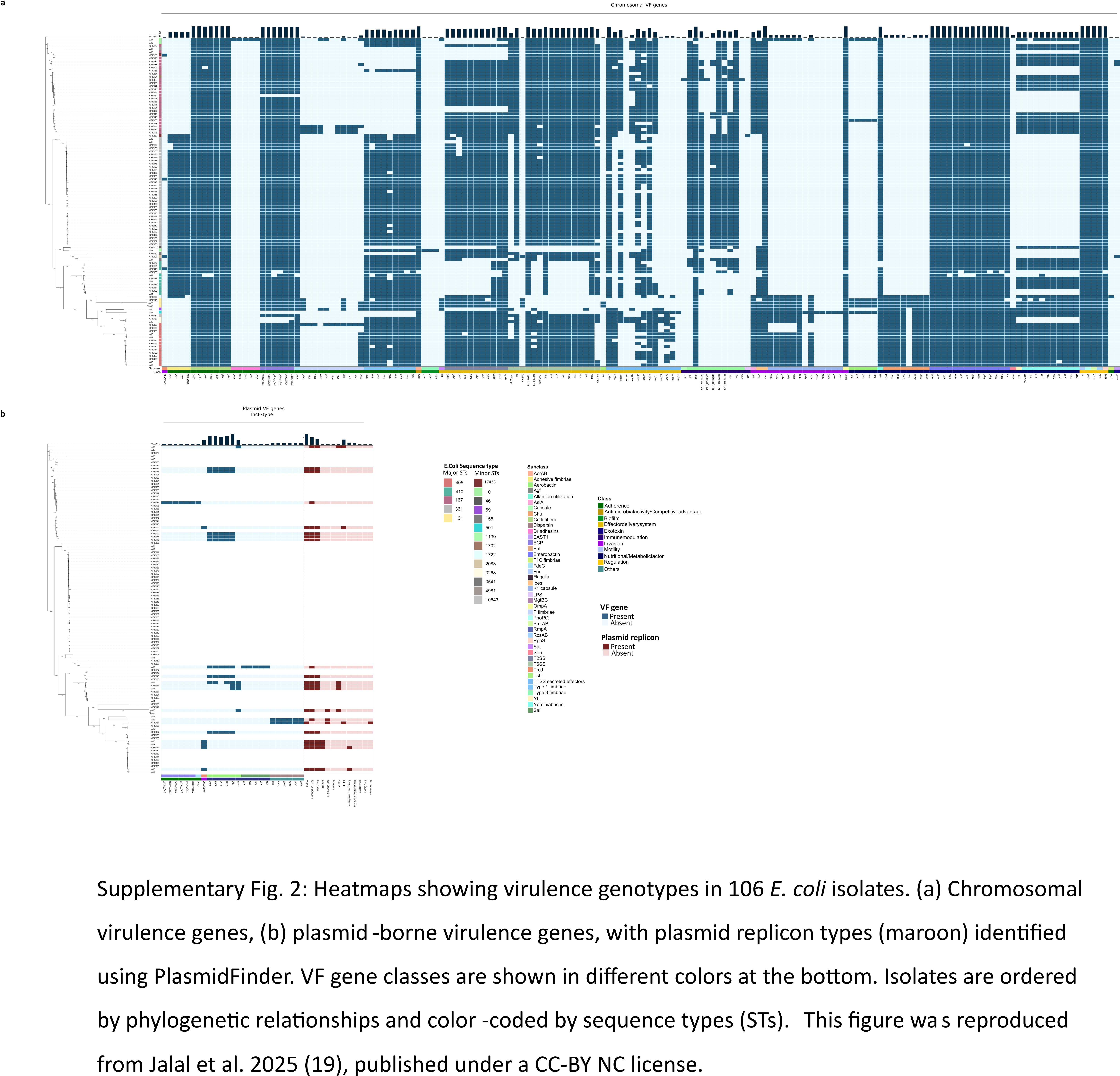

Supplement: Supplementary file 2 [file Image_2.jpg]

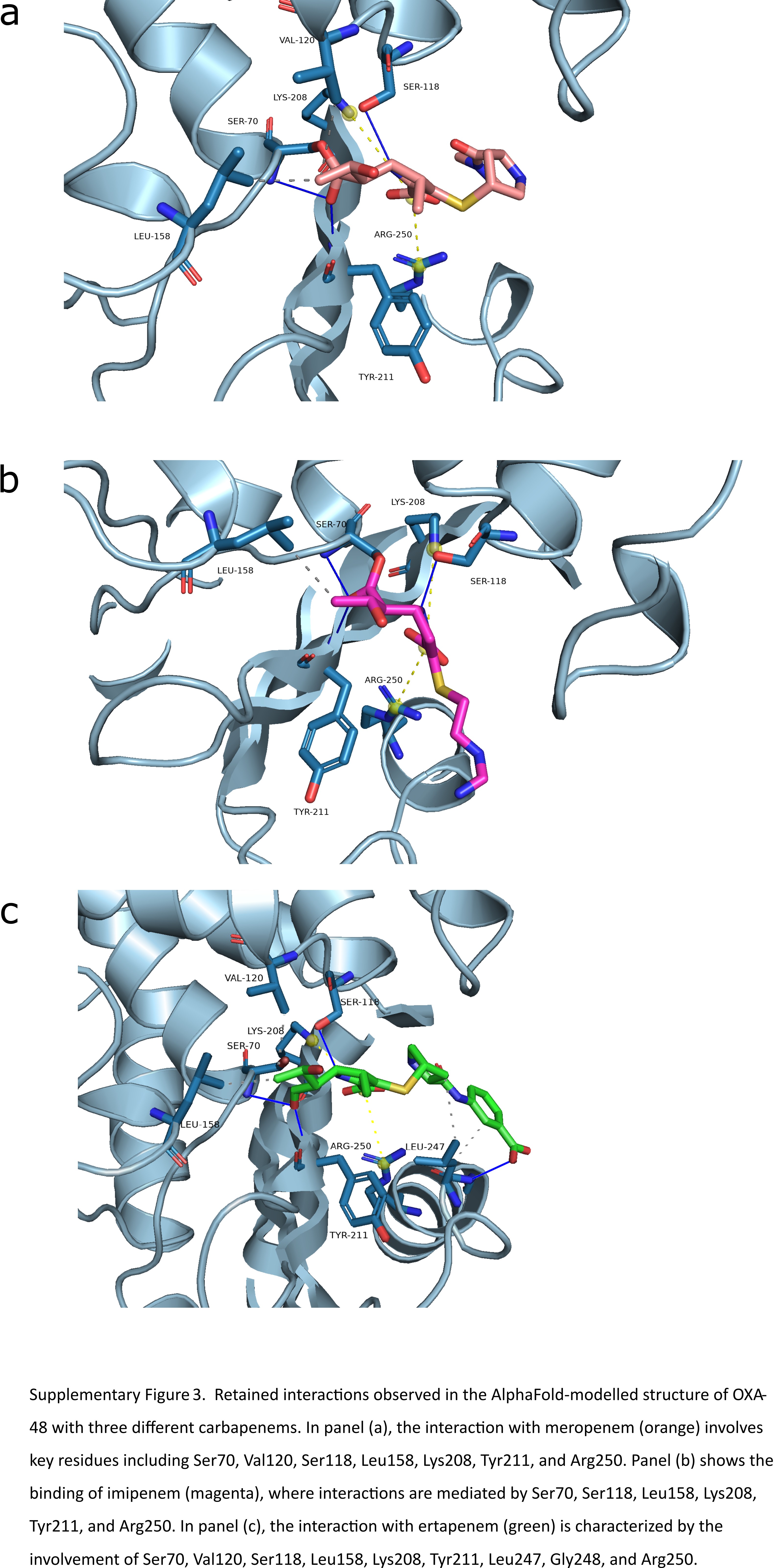

Supplement: Supplementary file 3 [file Image_3.jpg]
